# Evaluating the profound effect of gut microbiome on host appetite in pigs

**DOI:** 10.1186/s12866-018-1364-8

**Published:** 2018-12-14

**Authors:** Hui Yang, Ming Yang, Shaoming Fang, Xiaochang Huang, Maozhang He, Shanlin Ke, Jun Gao, Jinyuan Wu, Yunyan Zhou, Hao Fu, Congying Chen, Lusheng Huang

**Affiliations:** 1 State Key Laboratory of Pig Genetic Improvement and Production Technology, Jiangxi Agricultural University, Nanchang, 330045 China; 2 College of Bioscience and Engineering, Jiangxi Agricultural University, Nanchang, China; 3 National Engineering Research Center for Breeding Swine Industry, Guangdong Wens Foodstuff Co. Ltd., Xinxing, China

**Keywords:** Pig, Feed intake, Gut microbiota, Enterotype, 16S rRNA, Function prediction

## Abstract

**Background:**

There are growing evidences showing that gut microbiota should play an important role in host appetite and feeding behavior. However, what kind of microbe(s) and how they affect porcine appetite remain unknown.

**Results:**

In this study, 280 commercial Duroc pigs were raised in a testing station with the circadian feeding behavior records for a continuous period of 30–100 kg. We first analyzed the influences of host gender and genetics in porcine average daily feed intake (ADFI), but no significant effect was observed. We found that the *Prevotella*-predominant enterotype had a higher ADFI than the *Treponema* enterotype-like group. Furthermore, 12 out of the 18 OTUs positively associated with the ADFI were annotated to *Prevotella*, and *Prevotella* was the hub bacteria in the co-abundance network. These results suggested that *Prevotella* might be a keystone bacterial taxon for increasing host feed intake. However, some bacteria producing short-chain fatty acids (SCFAs) and lactic acid (e.g. *Ruminococcaceae* and *Lactobacillus*) showed negative associations with the ADFI. Predicted function capacity analysis showed that the genes for amino acid biosynthesis had significantly different enrichment between pigs with high and low ADFI.

**Conclusions:**

The present study provided important information on the profound effect of gut microbiota on porcine appetite and feeding behavior. This will profit us to regulate porcine appetite through modulating the gut microbiome in the pig industry.

**Electronic supplementary material:**

The online version of this article (10.1186/s12866-018-1364-8) contains supplementary material, which is available to authorized users.

## Background

Feelings of hunger and satiety are the principal involuntary motivations for animal feeding behavior [1]. The feeding behavior mainly includes two phases: the appetitive phase (e.g. foraging) and the consummatory phase (e.g. chewing and swallowing) [2]. The appetitive phase brings animals to contact with food, whereas the consummatory phase is the final reflexive response. Both of these phases can increase the hunger, the desire of appetite and food intake. Therefore, it is important to elucidate when to seek food, what to eat and how to eat. There are two models for appetite control: one is involved in host energy homeostasis and hedonics; the other model is based on bacteria–host communications [1].

The homeostatic model reflects the balance of energy intake and expenditure in the host. The model explains that appetite is triggered by energy shortage. The center for homeostatic control of energy balance is located in the hypothalamus, which plays a key role in integrating energy information through neural and humoral pathways [3]. The neural pathway senses peripheral energy status via vagal afferents, which relay the nutritional information to the solitary tract nucleus in the brainstem and subsequently transmits to the hypothalamus. The humoral pathway directly circulates nutrients and hormones (such as glucagon-like peptide 1 (GLP-1), peptide YY (PYY), insulin and leptin) to the hypothalamic arcuate nucleus (ARC). Within the hypothalamus, pro-opiomelanocortin (POMC) neurons regulate satiety, whereas neuropeptide tyrosine (NPY) and agouti-related peptide (AgRP) neurons are involved in hunger signaling. The POMC, NPY and AgRP neurons are often regarded as the ‘first order’ in the pathways of hunger and satiety [4]. When animals increase appetite and food intake, the feeding-related feeling of pleasure might underlie the hedonic reason for eating. An abnormal hedonic driving for eating can override homeostatic signals and cause eating disorders, such as bulimia nervosa [5] and obesity [6].

Accumulating evidences indicate that the gut microbiota plays an important role in the bidirectional communication between the brain and the gut [1, 7]. In particular, the microbiome in the gastrointestinal tract has an important influence in host energy metabolism. For instance, dysbioses of the gut microbiota has contributed to kwashiorkor [8], obesity [9] and anorexia nervosa [10]. More and more studies have indirectly demonstrated the role of the gut microbiota in host eating behavior. Zhang et al. [11] found that the gut microbiota could be shifted by changing the amount of food intake. Breton et al. [12] showed that gut bacterial proteins could activate host satiety pathways and have an effect on host control of food intake dependent on the bacterial growth cycles. The optimal regulation of host appetite might be ensured by both bacteria derived chemical signals and the energy status of the gut microbiota. The molecules produced by gut bacteria should be detected by the chemical sensory elements of the gut epithelium located on enteroendocrine cells, and then activate the pathways of appetite control [13]. However, these hypotheses about how the gut microbiota influences the host appetite remain uncovered. There are few studies about which microbes and how the gut bacteria regulate host feeding behavior. Furthermore, the causality between the change of the gut bacteria and host feeding behavior remains to be established.

The aim of the present research is to explore the potential impact of gut bacteria on porcine feeding behavior using 16S rRNA gene sequencing, and to identify the possible bacterial taxa influencing porcine appetite. As pigs have been used as a biomedical model for human diseases, the identification of bacterial molecules involved in feeding behavior could also provide useful reference for the prevention and treatment of human eating disorders.

## Results

### Phenotypic characteristics of porcine feeding behavior

**Fig. 1:**
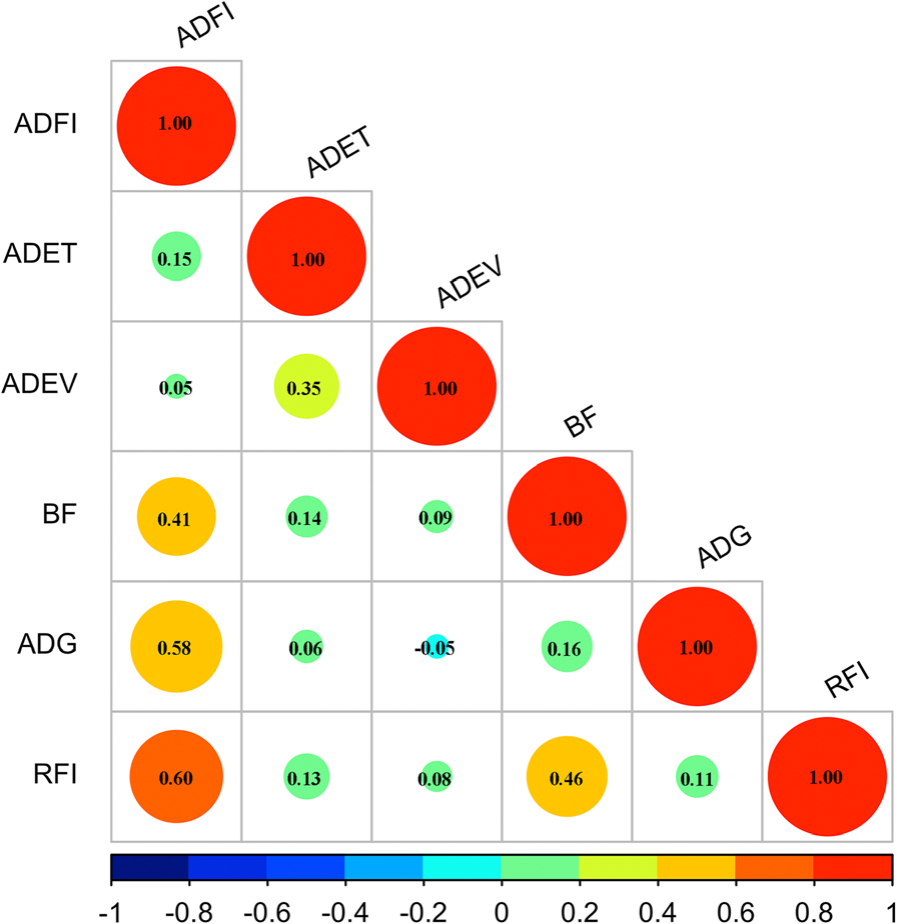
The correlation among six traits related to porcine feeding behavior. The numbers indicate spearman correlation coefficients between each pair of traits. The size of circle represents the correlation strength. The names of traits are shown as follows: average daily feed intake (ADFI), average daily eating time (ADET), average daily eating visits (ADEV), backfat (BF), average daily gain (ADG) and residual feed intake (RFI)

The phenotypic distributions of feeding behavior traits including average daily feed intake (ADFI), average daily eating time (ADET) and average daily eating visits (ADEV) in the experimental cohort are shown in Additional file 1: Figure S1. The phenotypic values basically fitted the normal distribution. As expected, the ADFI had a significantly positive correlation with backfat (*r* = 0.41), average daily gain (*r* = 0.58) and residual feed intake (*r* = 0.60), and the ADET was positively correlated with the ADEV. However, there was no significant correlation between ADFI and ADET, and between ADFI and ADEV (Fig. 1). We then analyzed the effects of gender, host genetics and pen on the phenotypical values. Host gender and genetics (full-sibs vs. unrelated individuals) showed no significant influences in the phenotypical values (*P* > 0.05, Additional file 2: Figure S2). However, pen had a significant effect on phenotypes. Pigs in different pens were detected to have significant differences of ADFI values by pairwise comparison (Additional file 3: Table S1). Besides, the pigs in the same pen exhibited more similarity of appetite (Additional file 2: Figure S2). Interestingly, our previous study also found that the diversity of gut microbiota in the pigs housed in the same pen was more similar than that in the pigs in different pens [14]. We assumed that the more similarity of gut microbial composition resulted in the similarity of appetite for pigs in the same pen.

### Enterotypes and its association with porcine feeding behavior

**Fig. 2:**
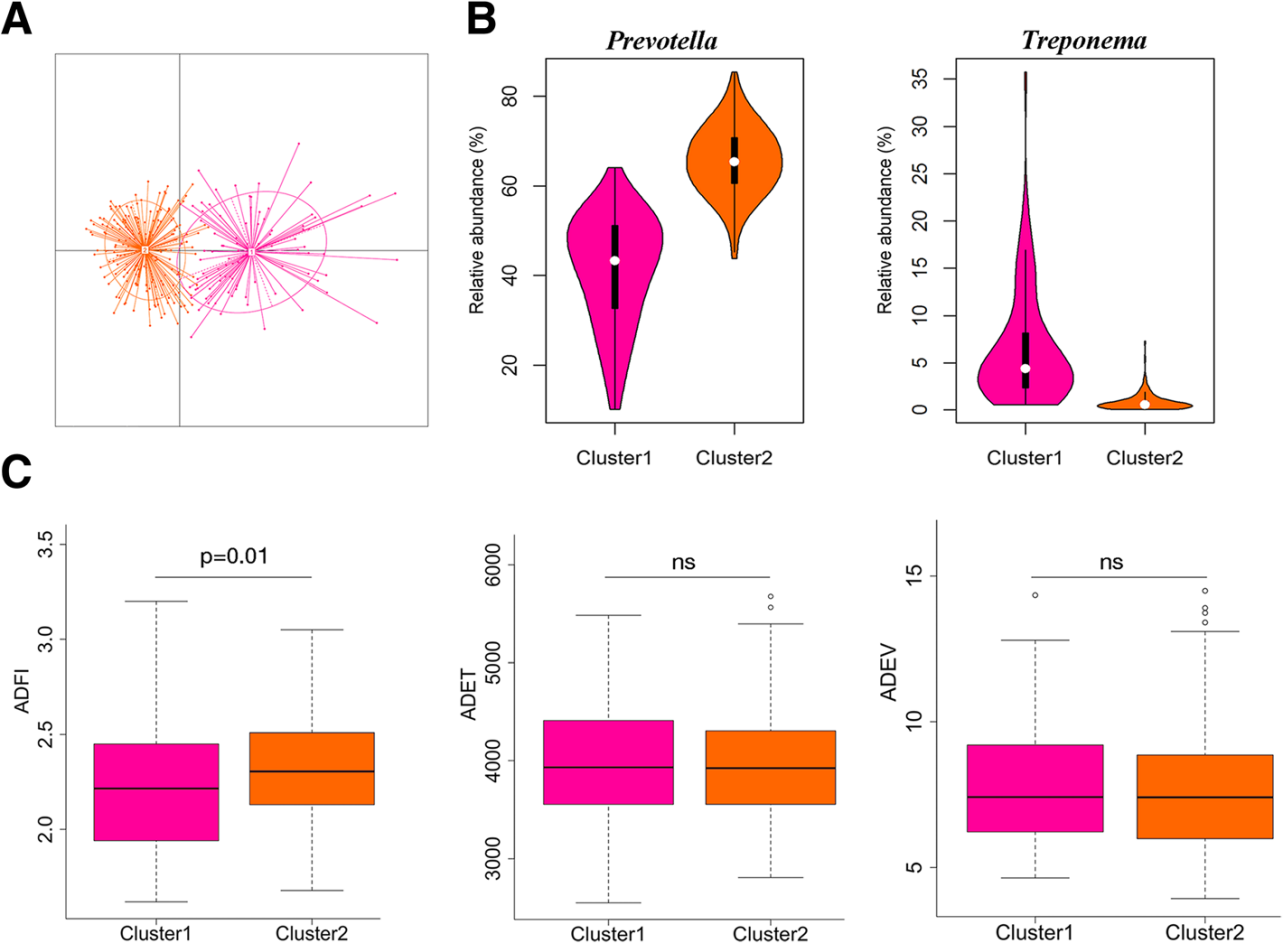
Enterotype-like cluster distribution in 280 experimental pigs. **a** Enterotype-like group assignation of experimental pigs. Enterotype 1 was dominated by *Treponema*, and enterotype 2 was dominated by *Prevotella*. **b** Relative abundance of *Treponema* and *Prevotella* in each enterotype. **c** Association of enterotype-like clusters with the ADFI, ADET and ADEV

We obtained 11,709,234 sequence reads in 280 fecal samples. After clustering based on the 97% similarity, an average of 759 OTUs for each sample was identified. Consistent with the previous reports [15, 16], Bacteroidetes and Firmicutes were the most abundant phyla in the fecal microbiota of pigs. But significant difference existed in the relative abundances of bacterial taxa among 280 samples, suggesting the animal-to-animal variation of the phylogenetic composition. We classified the enterotypes for all tested samples according to their phylogenetic composition via the method described by Arumugam et al. [17]. PCA analysis was used to observe the enterotype patterns of stool samples. Two enterotypes were identified, which were dominated by *Treponema* (enterotype 1) and *Prevotella* (enterotype 2), respectively (Fig. 2a). The violin plots illustrated the relative abundances of dominant bacteria between the two enterotypes (Fig. 2b). In addition, the relationship of the enterotypes with porcine feeding behavior traits is shown in Fig. 2c. The *Prevotella*-predominant enterotype (cluster 2) had a significantly higher ADFI value than the *Treponema*-enterotype (*P* = 0.01). However, the enterotype-like clusters were not significantly associated with the ADET or ADEV (*P* > 0.05, Fig. 2c).

### Association of fecal bacteria with porcine feeding behavior

**Fig. 3:**
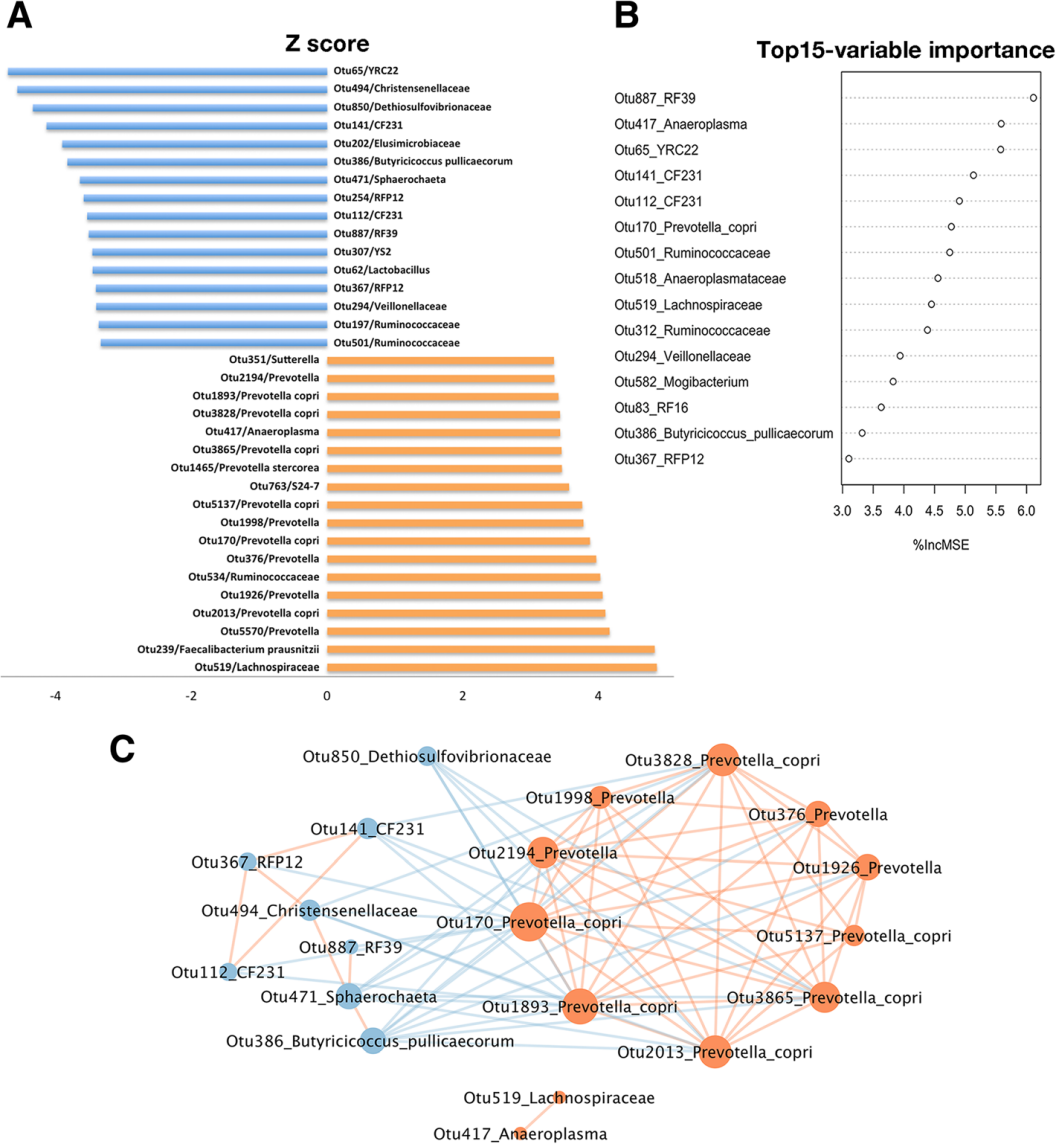
Porcine average daily feed intake (ADFI)-associated OTUs identified in this study and its interaction network. **a** The 34 ADFI-associated OTUs identified by two-part model (FDR < 0.05) are shown as Z scores. Blue bars show the negative associations and orange bars indicate the positive associations. **b** The top informative OTUs related to the ADFI detected by randomForest analysis. **c** Co-abundance network analysis of the ADFI-associated OTUs reveals the significant interactions. The orange nodes correspond to the OTUs showing the positive association with the ADFI, and the blue nodes represent the OTUs negatively associated with the ADFI. Edge color indicates positive (orange) and negative (blue) correlations. The size of nodes represents the degree of connectivity

A total of 34 OTUs were significantly associated with the ADFI in the two-part model analysis (FDR < 0.05, Fig. 3a). Eighteen out of the 34 OTUs had strongly positive correlations with the ADFI, while the other 16 OTUs showed significantly negative associations (Fig. 3a and Additional file 4: Table S2). Of the 18 positive associations, 12 OTUs were annotated to *Prevotella*, and the other six OTUs were separately assigned to *Lachnospiraceae*, *Faecalibacterium prausnitzii*, *Ruminococcaceae*, *S24–7*, *Anaeroplasma* and *Sutterella*. Among the 16 negative correlations, eight OTUs were annotated to the family level, including two OTUs annotated to *Ruminococcaceae*, two OTUs to *RFP12*, and one OTU to each of *Christensenellaceae*, *Dethiosulfovibrionaceae*, *Elusimicrobiaceae* and *Veillonellaceae*. Five OTUs were assigned to the genus level, including two OTUs to *CF231*, and the other three OTUs to *Sphaerochaeta*, *YRC22* and *Lactobacillus*. Only OTU386 was annotated to the species *Butyricicoccus pullicaecorum.* However, at the threshold of FDR < 0.05, we didn’t detect any OTUs significantly associated with the ADET or ADEV.

We also employed randomForest for regression analysis to further evaluate the association between fecal bacteria and the ADFI. A total of 15 OTUs were identified to significantly associate the ADFI. These OTUs were annotated to the bacterial taxa similar to those identified by two-part model, suggesting the repeatability of the association results (Fig. 3b). Additionally, we also identified two OTUs for each of the ADET and ADEV by randomForest analysis (Additional file 5: Figure S3).

To determine the hub OTUs related to the ADFI, the 34 OTUs detected by the two-part model were used to estimate sparse correlations using the SparCC method [18]. Only OTU170 (*Prevotella copri*) had the connectivity with all other nodes (Fig. 3c). We also observed that those OTUs positively associated with the ADFI and annotated to *Prevotella* were clustered into a sub-module that was negatively correlated with the OTUs showing negative effect on the ADFI. Combining the results of the association study and the co-abundance network analysis, we suggested that *Prevotella*, especially *Prevotella copri* should play a core effect on porcine feed intake.

### Contribution of the gut bacteria to the pen effect on the ADFI

To test the hypothesis that the pen effect on the ADFI values might be associated with the stochastic differences in the bacterial exchange and colonization between pigs in the same pen and different pens, we focused on those OTUs that belonged to the 34 ADFI-associated OTUs and showed significant difference of the relative abundances between pigs in different pens. As the result, the relative abundances of five ADFI-associated OTUs (Otu351, Otu494, Otu202, OTU367 and Otu294) were significantly influenced by pens (Additional file 6: Table S3). We next treated the relative abundance of the OTU351, OTU494 and OTU202 as the fixed effect in the linear regression analysis, and re-evaluated the effect of pens on the ADFI values. Interestingly, the significant difference of the ADFI values in pigs in different pens was indeed vanished (*P* = 0.07), suggesting that the large variation of the phylogenetic composition of gut microbiota in pigs housed in different pens caused the pen effect on the ADFI.

### Predicted function capacities of gut microbiome related to porcine ADFI

**Fig. 4:**
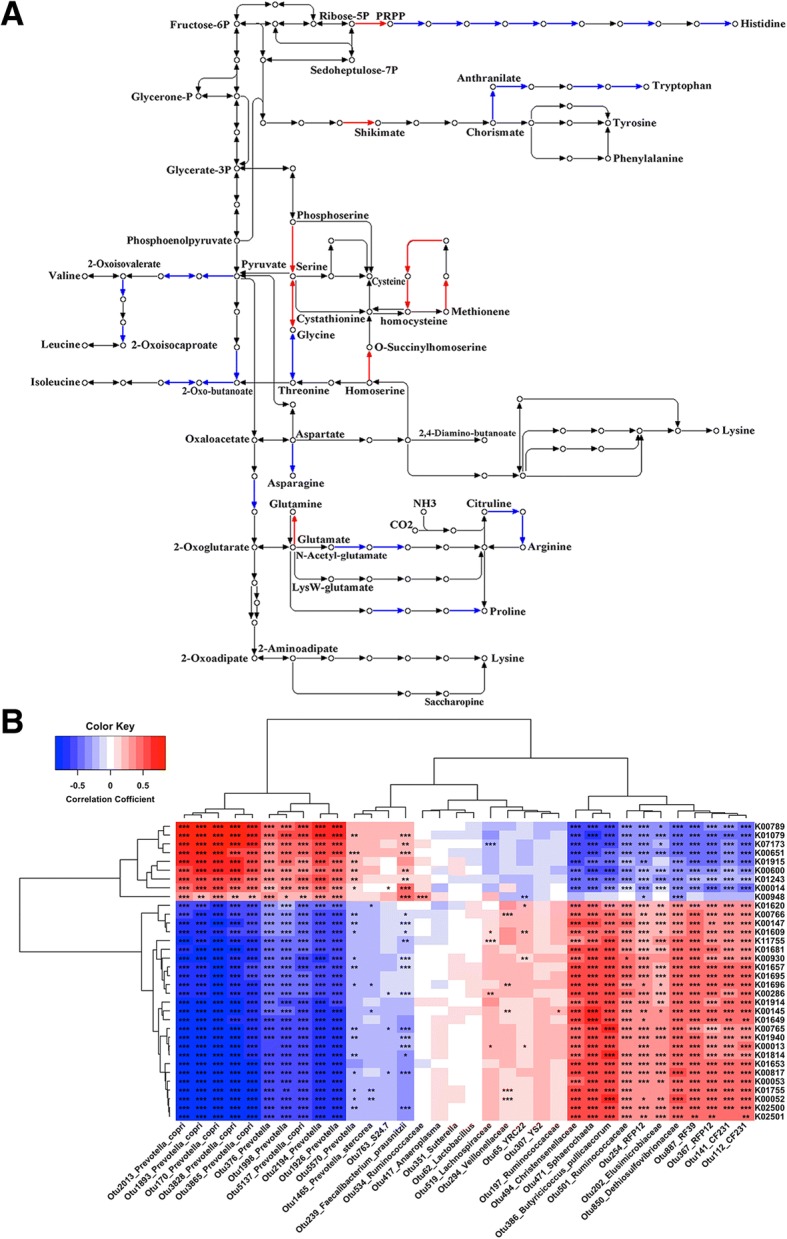
The amino acid biosynthesis pathway enriched by the ADFI-associated KEGG Orthologies (KO) and its correlation with the ADFI-associated OTUs. **a** The amino acid biosynthesis pathway enriched by the ADFI-associated KOs. Red lines represent positive correlations with the ADFI, while blue lines indicate negative correlations with the ADFI. **b** The correlation between the ADFI-associated OTUs and the ADFI-associated KOs related to amino acid metabolism. * *P* < 0.05, ** *P* < 0.01 and *** *P* < 0.001. The definitions of KOs are listed in Additional file 7: Table S4

We predicted functional capacity profiling using 16S rRNA marker gene sequences to identify potential function categories of gut microbiome related to porcine ADFI by PICRUST software [19]. At the significance threshold of FDR < 0.05, we identified 204 KEGG Orthologies (KOs) showing significant associations with the ADFI (Additional file 7: Table S4). These ADFI-associated KOs were enriched in the pathways related to amino acid biosynthesis and metabolism (cysteine and methionine metabolism, histidine metabolism, phenylalanine, tyrosine and tryptophan biosynthesis, and alanine, aspartate and glutamate metabolism), and sugar metabolism (such as amino sugar and nucleotide sugar metabolism, starch and sucrose metabolism, and fructose and mannose metabolism) (Additional file 8: Figure S4). Interestingly, the KOs negatively associated with the ADFI were enriched in the pathways of the biosynthesis of branched chain amino acids (valine, leucine and isoleucine), histidine, tryptophan, arginine and proline, while the KOs positively associated with the ADFI were enriched in the biosynthesis of serine, cysteine and methionine (Fig. 4a). We further evaluated the contribution of the ADFI-associated bacterial taxa to the changes of the ADFI-associated pathways related to amino acid biosynthesis and metabolism. Those bacterial taxa increasing porcine ADFI, especially *Prevotella* and *Faecalibacterium prausnitzii*, were positively associated with the KOs enriched in the biosynthesis of serine, cysteine and methionine, but negatively correlated with those KOs negatively associated with the ADFI (*P* < 0.001). On the contrary, the bacterial taxa decreasing porcine ADFI showed the inverse correlations with the ADFI-associated KOs (Fig. 4b).

## Discussion

The role of gut microbiota in the regulation of host physiological functions in both health and disease state is a hot topic in the research field of gut microbiome. Researchers have summarized the possible role of gut microbiota in host appetite control [1] and the mechanism of the microbiota-gut-brain axis in regulating host appetite and metabolism [7]. Furthermore, the correlation between the diversity of the gastrointestinal microbial community and cattle feed intake was also investigated [20]. However, to our knowledge, there is no study identifying gut microbes associated with porcine appetite until now. In the present study, we uncovered the potential relationships between porcine feeding behavior traits including average daily feed intake, average daily eating visits and average daily eating time, and gut bacterial taxa by 16S rRNA gene sequencing analysis. We also revealed potential function capacities of gut microbiome related to porcine ADFI.

The ADFI was positively associated with backfat thickness, average daily gain and residual feed intake, suggesting that increasing feed intake not only promotes growth, but also increases fat deposition, but possibly decreases feed efficiency. A total of 34 OTUs were identified to associate porcine ADFI, but we did not identify any OTUs associated with the ADET or ADEV in this study. Moreover, the phenotypic values of eating visits and eating time were not correlated with feed intake. The main explanation for this observation should be the reason that it was difficult to accurately record porcine eating time and visiting times, because some pigs did not intake the feed when they visited the automatic feeding trough or the pigs only felt funny for out and in of the machine.

Some SCFA-producing bacteria showed negative correlations with feed intake (Fig. 3a). For examples, *Christensenellaceae* (OTU494) is Gram-negative bacteria, and can produce the SCFAs [21]. Goodrich et al. [22] revealed that *Christensenellaceae* might be a marker for low BMI in humans. This was concordance with the finding in this study that the ADFI was positively associated with backfat thickness. *Butyricicoccus pullicaecorum* (OTU386) is an anaerobic and butyrate-producing bacterium [23]. *Veillonellaceae* (OTU294) that contains the genus *Mitsuokella*, *Megasphaera* and *Acidaminococcus,* could utilize dietary amino acids [24] and produce high amounts of the SCFAs [25]. Higher abundance of *Ruminococcaceae* (OTU197 and OTU501) was found in the low-ADFI pigs. Some species of *Ruminococcaceae* (e.g. *Ruminococcus flavefaciens* and *Ruminococcus albus*) can produce lactate and propionate [26]. SCFAs have an important effect on regulating host energy metabolism and appetite. Locally in the gut, SCFAs can directly facilitate the release of the anorexigenic hormones PYY and GLP-1 from L-cells [27]. Chambers et al. [28] showed that there was an increase in the hormones PYY and GLP-1, and a decrease in energy intake when delivering propionate directly to the colon. In addition,* Lactobacillus* (OTU62) was detected to negatively associate the ADFI in this study. *Lactobacillus* has always been used as probiotics and can produce lactic acid to promote intestinal development and metabolism [29]. High level of lactate can activate the satiation pathway and reduce food intake [30]. Pessione et al. [31] summarized that biogenic amines from lysine, phenylalanine, histidine and tyrosine are released by decarboxylation of lactic acid bacteria. Correspondingly, in the predicted function capacity analysis of gut microbiome based on 16S rRNA gene sequencing data, the genes involved in the metabolism and biosynthesis of histidine, arginine, proline, branched chain amino acids, and aromatic amino acids (tryptophan, phenylalanine and tyrosine) were increased along with the declining feed intake. The activity of histamine neurons could control energy balance and behavioral responses. Interestingly, feeding a low protein diet added with lysine, methionine and tryptophan to piglets reduced feed intake and body weight gain [32]. Batterham et al. [33] also reported that protein inhibits food intake and appetite more than carbohydrate or fat in obese individuals. The increased concentration of branched amino acid in the plasma significantly increased the release of some gut hormones (e.g. GLP-1 and insulin) [34]. Oral administration of phenylalanine reduced food intake in diet-induced obese mice [35]. Especially, tryptophan significantly increased plasma concentrations of PYY and cholecystokinin that are both regarded as satiation signal [36]. Holzer [37] indicated that the synthesis of neuropeptides might be affected by microbial control of the availability of amino acids. These results suggested that some gut bacteria might suppress porcine feed intake through producing SCFAs and lactic acid, and regulating the metabolism of amino acids. However, compared to the functional prediction based on 16S rRNA sequencing data, a follow-up metagenomic sequencing analysis in the future study would help better to elucidate the function and mechanism of gut microbiota suppressing feed intake.

In agreement with the previous report [38], *Prevotella* was the hub in the interaction network of the ADFI-associated OTUs. In the enterotype analysis, we identified that the *Prevotella*-enterotype had a greater ADFI than the *Treponema* enterotype-like cluster. Myer et al. [20] also showed that *Prevotella* had a higher abundance in the high ADFI group than in the low ADFI individuals in beef cattle. A previous report indicated that *Prevotella* is in connection with an increased long-term carbohydrate intake [39]. *Prevotella* that is capable of metabolizing complex dietary polysaccharides may favor the uptake of monosaccharides in the host gut [38]. The ADFI-associated OTUs annotated to *Prevotella* were significantly associated with the ADFI-associated KOs. Queipo-Ortuno et al. [40] reported that the abundance of *Prevotella* was positively correlated with serum ghrelin which is the only appetizing hormone known at present. Taken together, we speculated that *Prevotella* (particularly *Prevotella copri*) might be a critical bacterial taxon stimulating the feed intake.

## Conclusion

The present study showed that some bacteria producing SCFAs and lactic acid (e.g. *Ruminococcaceae* and *Lactobacillus*) might play an important role in suppressing porcine feed intake, while *Prevotella* could promote porcine feed intake and might be the keystone bacteria for host appetite control. These results suggested that the gut microbial community might have an important contribution to porcine feeding behavior. The modulation of gut microbiota could be benefit for the control of feed intake in pig industry.

## Methods

### Experimental animals and phenotypic measurement

A total of 280 Duroc pigs (111 females and 169 males) were used in this study. All experimental pigs were healthy and not received antibiotics, probiotics or prebiotics during the period of experiment. Feeding and management of all experimental pigs were described in detail in our previous study [14]. Briefly, all experimental pigs were weaned at the age of 28 days, and then raised in the nursery house until its body weight achieved 30 kg. After that, experimental pigs were transferred to the fattening house where automatic feeding troughs (Osborne Industries, USA) were installed. Male and female pigs were separately housed in different pens. The same formula feed and clean water were available ad libitum for all experimental pigs. Phenotypic performances of experimental pigs at the stage of fattening from 30 kg (at the age of 70 ~ 90 days) to 100 kg body weight (at the age of 170 ~ 190 days) were measured by the instrument of automatic feeding trough (Osborne Industries, USA)*.* When their body weight achieved 100 kg, the experimental pigs were slaughtered at a commercial slaughterhouse by bleeding after electrical stunning. To avoid the effect of environmental adaptation of experimental pigs with automatic feeding trough, we used the phenotypic values obtained from day 100 to 160 (intermediate stage of measurement) for further analysis. ADFI, ADET and ADEV were used as the traits evaluating feeding behavior and appetite. As the phylogenetic composition and microbial ecosystem of gut microbiota remain relatively stable after maturation [41], we collected the fecal samples from all 280 experimental pigs at the age of 140 days (within the interval of 100–160 days). All fecal samples were harvested from animal’s anus, and then immediately dipped in liquid nitrogen. After transported to the laboratory, the samples were transferred into -80 °C freezer until use.

### Evaluating the environmental and host effects on phenotypic values of feeding behavior

The effects of pen and host kinship on phenotypic values of feeding behavior were evaluated by comparing the similarity of phenotypic values between the pigs in the same pen or in the full-sib members, and the pigs in different pens or in unrelated individual groups. *T*-test was used to compare the phenotypic values between male and female pigs.

### 16S rRNA gene sequencing and data processing

Microbial DNA was extracted from feces with QIAamp Fast DNA Stool Mini Kit (Qiagen, Germany) according to the manufacturer’s protocol. The V4 region of the 16S rRNA gene was amplified using the barcode fusion primers 515F (5’-GTGCCAGCMGCCGCGGTAA-3′) and 806R (5’-GGACTACHVGGGTWTCTAAT-3′). The PCR products were used to construct the libraries, and then sequenced using the paired-end method on Illumina MiSeq platform (Illumina, USA). Data processing was performed by the standard protocols of bioinformatics analysis for 16S rRNA gene sequencing. Firstly, the primers, barcode sequences, and low quality reads were removed from raw data [42]. High-quality paired-end clean sequence reads were then assembled into tags using FLASH (v.1.2.11). And then, USEARCH software (v7.0.1090) was used to pick OTUs at 97% similarity [43]. Taxonomic assignments for the aligned sequences were made using the Ribosomal Database project (RDP) classifer program (v2.2) [44]. Those OTUs that were presented in less than 5% of the experimental pigs and had relative abundance < 0.01% were removed from further analysis.

### Enterotype analysis

According to the method described by Arumugam et al. [17], we analyzed the enterotype of the experimental pig cohort. In brief, the clustering of samples was performed by using the *Partitioning Around Medoids* (PAM) clustering algorithm and *Jensen-Shannon* divergence (JSD) distance based on the relative abundances of bacteria at the genus level. And then, the *Calinski-harabasz* (CH) Index was used to evaluate the optional number of clusters from 2 to 20 clusters. When the cluster number had the highest CH index and was also validated by the silhouette coefficient, it was set as the optimal number. The possible effect of predicted enterotype-like clusters on the host feeding behavior was tested by *t-*test.

### Association study

Because of the non-normal distribution of the relative abundances of most bacteria in the experimental pigs, a two-part model was applied to analyze the association of bacteria with the traits of feeding behavior as used in previous study [45]. Briefly, the two-part model accounts for both binary and quantitative features. The binary analysis was to test for the effect of the presence/absence of the microbes on porcine feeding behavior, and the quantitative model analyzed for association between the abundance of the detected microbes and the host feeding behavior. To further evaluate the effect of both binary and quantitative features, a meta-analysis was performed using an unweighted Z method. The minimum *P*-value of binary, quantitative and meta-analysis was set as the final association *P*-value. The Z-score was calculated based on the Z distribution. In addition, 1000 × permutation test was used to control the false discovery rate (FDR). The FDR ≤ 0.05 was set as the significant threshold. The residuals of ADFI, ADET and ADEV values corrected the effect of sex were used for association analysis. We also used the package of randomForest for regression to further confirm the association results [46]. The variable importance was estimated using the index of increase in Mean Squared Error (%incMSE). The %incMSE value above 3 was considered as an important variable.

### Network analysis for the ADFI-associated OTUs

To identify the hub OTUs from the ADFI-associated OTUs detected by two-part model, OTU networks were predicted using the SparCC approach as described by Friedman et al. [18]. In brief, we first calculated the SparCC correlation coefficient matrix for the ADFI-associated OTUs with SpiecEasi package [47]. And then, the network analysis was performed and visualized using cytoscape at the absolute correlation coefficient threshold of 0.3 [48].

### Prediction of functional capacity of gut microbiome

To predict functional capacity of the gut microbiome, PICRUST (v1.0.0) was applied to calculate the relative abundances of KEGG pathways according to the 16S rRNA gene sequences [19]. Correlations between ADFI values and relative abundances of KOs were implemented with MaAsLin. The association between the ADFI-associated OTUs detected by two-part model and the ADFI-related KOs was evaluated by spearman correlation analysis with R software.

## Additional files

Additional file 1: **Figure S1.** Distribution of phenotypic values of porcine feeding behavior-related traits. The phenotypic values of feeding behavior-related traits were basically in a normal distribution. The name of each trait was shown on the top: Average daily feed intake (ADFI), average daily eating time (ADET) and average daily eating visits (ADEV) (TIF 1374 kb)

Additional file 2: **Figure S2.** Factors affecting porcine feeding behavior traits. (A) Sex had no significant effect on porcine feeding behavior traits. (B) Pen showed significant effect on porcine feeding behavior traits. (C) Comparison of porcine feeding behavior traits between full-siblings and unrelated individuals (ns: *P* value was not achieved significance; *** *P* < 0.001 for student’s t-test). (TIF 1241 kb)

Additional file 3: **Table S1.** The significant differences of ADFI values between pigs in different pens detected by pairwise comparison. (XLS 30 kb)

Additional file 4: **Table S2.** The OTUs significantly associated with the ADFI by the two-part model (FDR < 0.05). (XLS 44 kb)

Additional file 5: **Figure S3.** The top informative OTUs for ADET (A) and ADEV (B) detected by randomForest analysis. (TIF 529 kb)

Additional file 6: **Table S3.** Five OTUs out of the 34 ADFI-associated OTUs whose relative abundances were significantly influenced by pens. (XLS 33 kb)

Additional file 7: **Table S4.** The correlation between the relative abundance of KOs and the ADFI by MaAsLin. (XLS 65 kb)

Additional file 8: **Figure S4.** The top 25 pathways enriched by the ADFI-associated KEGG Orthologies (KOs). The data on the bars indicates the KO numbers. (TIF 2160 kb)

Additional file 9: **Table S5.** The ARRIVE Guidelines Checklist. (DOC 59 kb)

## Abbreviations

ADET: Average daily eating time
ADEV: Average daily eating visits
ADFI: Average daily feed intake
ADG: Average daily gain
AgRP: Agouti-related peptide
ARC: Hypothalamic arcuate nucleus
BF: Backfat
CH: Calinski-harabasz index
FDR: False discovery rate
GLP-1: Glucagon-like peptide 1
JSD: Jensen-Shannon divergence distance
KEGG: Kyoto Encyclopedia of Genes and Genomes
MSE: Mean squared error
NPY: Neuropeptide tyrosine
OTU: Operational taxonomic units
PAM: Partitioning around medoids
POMC: Proopiomelanocortin
PYY: Peptide YY
RFI: Residual feed intake
rRNA: Ribosomal Robonucleic Acid
SCFA: Short-chain fatty acid
