# Supplementary figures and images for: Evaluating the profound effect of gut microbiome on host appetite in pigs
Source: BMC Microbiol. 2018 Dec 14;18:215. doi: 10.1186/s12866-018-1364-8 (PMC6295093; doi:10.1186/s12866-018-1364-8)

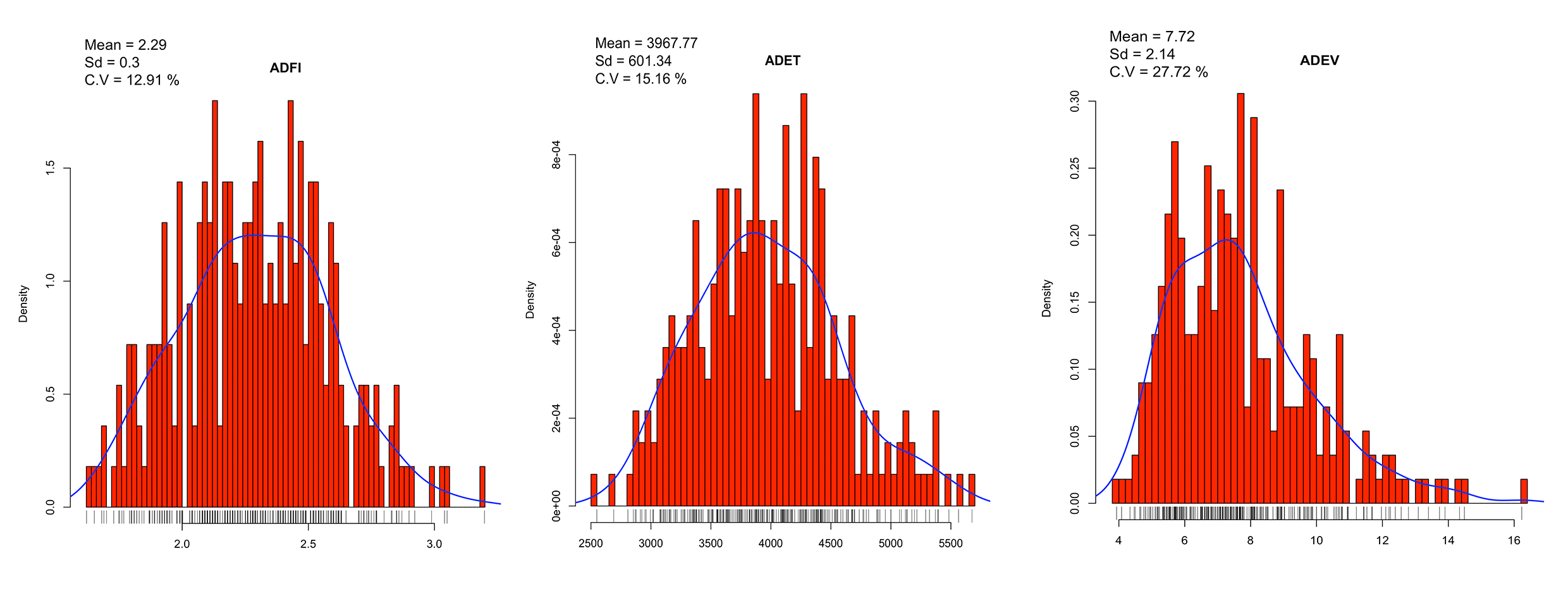

Supplement: Supplementary file 1 — Figure S1. Distribution of phenotypic values of porcine feeding behavior-related traits. The phenotypic values of feeding behavior-related traits were basically in a normal distribution. The name of each trait was shown on the top: Average daily feed intake (ADFI), average daily eating time (ADET) and average daily eating visits (ADEV) (TIF 1374 kb) [file 12866_2018_1364_MOESM1_ESM.tif]

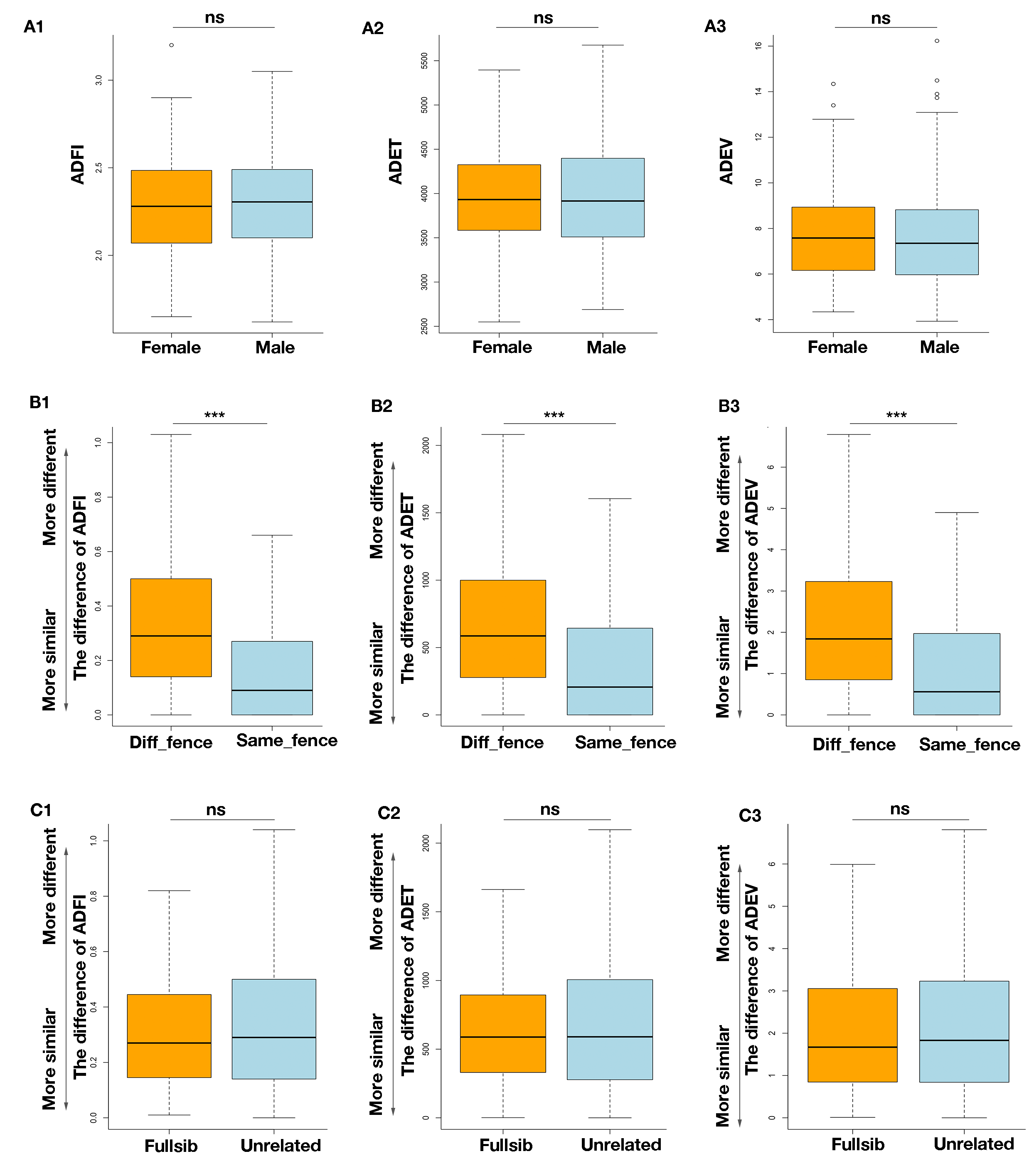

Supplement: Supplementary file 2 — Figure S2. Factors affecting porcine feeding behavior traits. (A) Sex had no significant effect on porcine feeding behavior traits. (B) Pen showed significant effect on porcine feeding behavior traits. (C) Comparison of porcine feeding behavior traits between full-siblings and unrelated individuals (ns: P value was not achieved significance; *** P < 0.001 for student’s t-test). (TIF 1241 kb) [file 12866_2018_1364_MOESM2_ESM.tif]

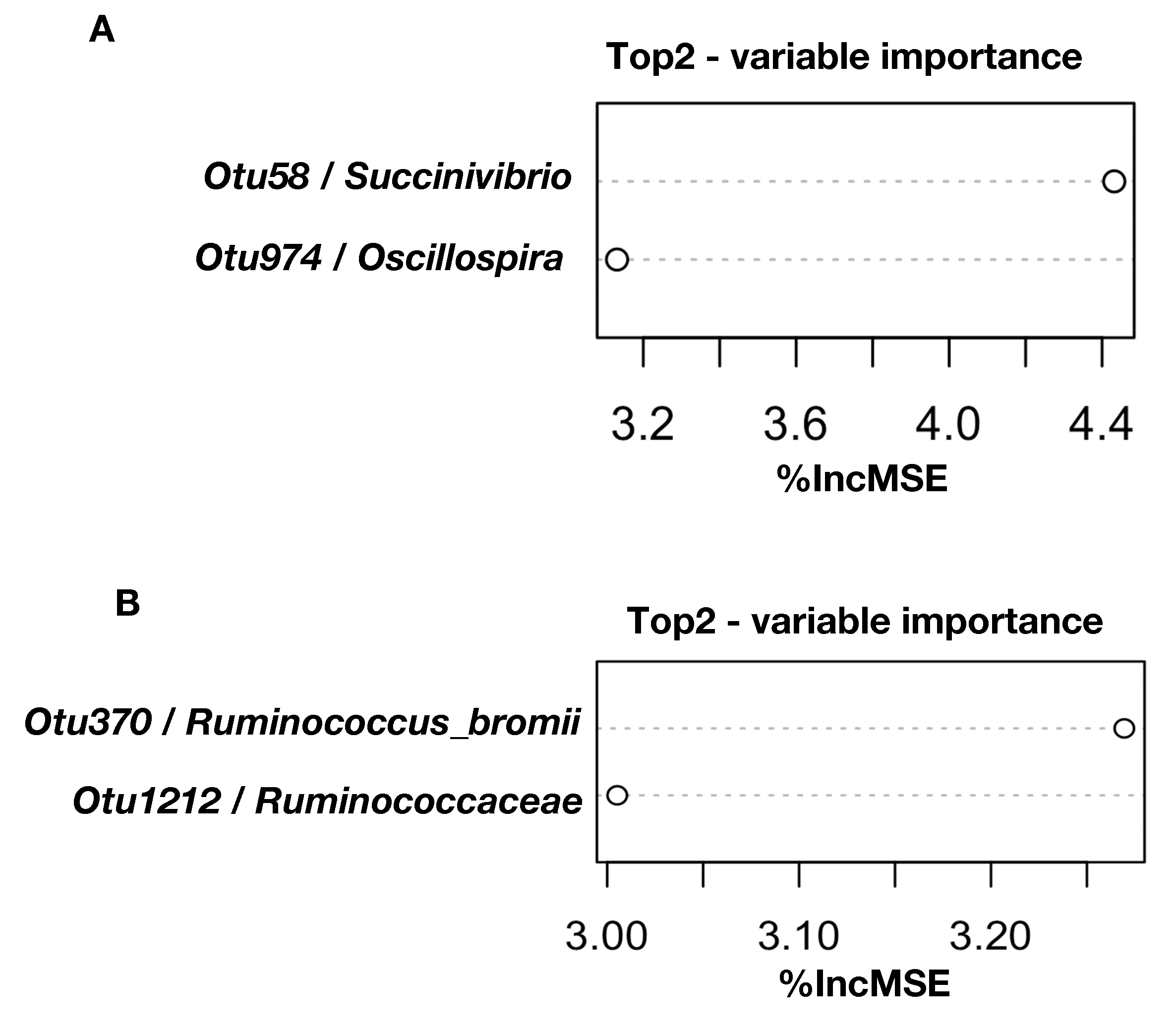

Supplement: Supplementary file 5 — Figure S3. The top informative OTUs for ADET (A) and ADEV (B) detected by randomForest analysis. (TIF 529 kb) [file 12866_2018_1364_MOESM5_ESM.tif]

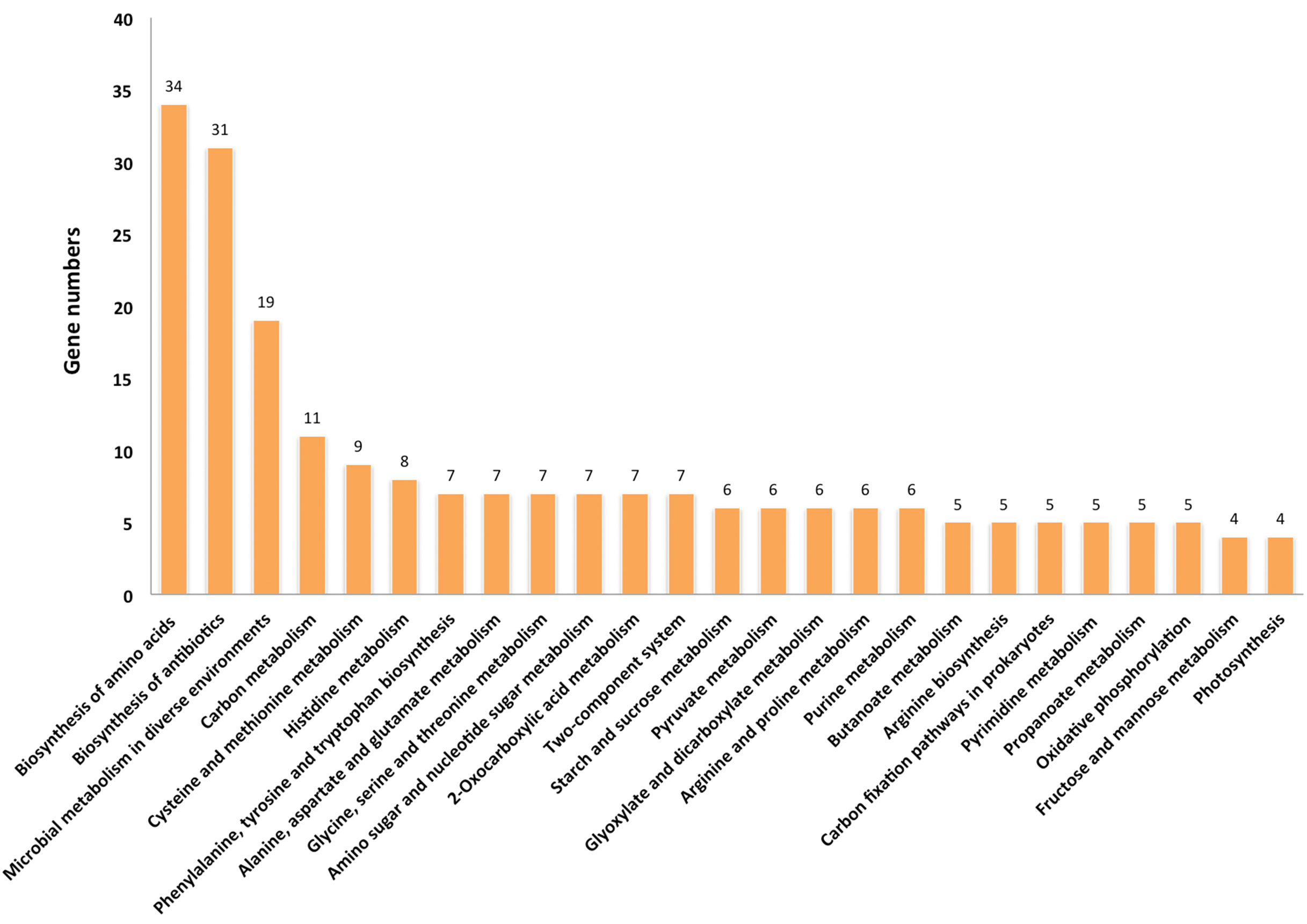

Supplement: Supplementary file 8 — Figure S4. The top 25 pathways enriched by the ADFI-associated KEGG Orthologies (KOs). The data on the bars indicates the KO numbers. (TIF 2160 kb) [file 12866_2018_1364_MOESM8_ESM.tif]
