# Supplementary material for: Evaluating the profound effect of gut microbiome on host appetite in pigs
Source: BMC Microbiol. 2018 Dec 14;18:215. doi: 10.1186/s12866-018-1364-8 (PMC6295093; doi:10.1186/s12866-018-1364-8)
Supplement: Supplementary file 9 — Table S5. The ARRIVE Guidelines Checklist. (DOC 59 kb) [file 12866_2018_1364_MOESM9_ESM.doc]

**Additional file 9: Table S5** The ARRIVE Guidelines Checklist.

|  | ITEM | RECOMMENDATION |
| --- | --- | --- |
| **TITLE** | 1 | Evaluating the profound effect of gut microbiome on host appetite in pigs |
| **ABSTRACT** | 2 | **Background**: There are growing evidences showing that gut microbiota should play an important role in host appetite and feeding behavior. However, what kind of microbe(s) and how they affect porcine appetite remain unknown.  **Results:** In this study, 280 commercial Duroc pigs were raised in a testing station with the circadian feeding behavior records for a continuous period of 30-100 kg. We first analyzed the influences of host gender and genetics in porcine average daily feed intake (ADFI), but no significant effect was observed. We found that the *Prevotella*-predominant enterotype had a higher ADFI than the *Treponema* enterotype-like group. Furthermore, 12 out of the 18 OTUs positively associated with the ADFI were annotated to *Prevotella*, and *Prevotella* was the hub bacteria in the co-abundance network. These results suggested that *Prevotella* might play a key role in increasing host feed intake. However, some bacteria producing short-chain fatty acids (SCFAs) and lactic acid (e.g. *Ruminococcaceae* and *Lactobacillus*) showed negative associations with the ADFI. Predicted function capacity analysis further identified that the genes for amino acid metabolism showed significantly different enrichment between pigs with high and low ADFI value.  **Conclusions:** This study showed that some bacteria producing SCFAs and lactic acid might suppress the host feed intake, while *Prevotella* might have a major effect on stimulating the feed intake. The present research provided important information on the profound effect of gut microbiota on porcine appetite and feeding behavior. |
| **INTRODUCTION** |  |  |
| **Background** | 3 | There are growing evidences showing that gut microbiota should play an important role in host appetite and feeding behavior. However, what kind of microbe(s) and how they affect porcine appetite remain unknown. |
| **Objectives** | 4 | The primary objectives of the study: a) to explore the potential impact of gut bacteria on porcine feeding behavior using 16S rRNA gene sequencing, b) and to identify the possible bacterial taxa influencing porcine appetite. |
| **METHODS** |  |  |
| **Ethical statement** | 5 | All animal works were conducted according to the guidelines for the care and use of experimental animals established by the Ministry of Agriculture of China. The project was approved by Animal Care and Use Committee (ACUC) in Jiangxi Agricultural University. |
|  |  |  |
| **Study design** | 6 | A total of 280 commercial Duroc pigs (111 females and 169 males) were used in this study. The experimental pig cohort contained 24 pairs of half-siblings and 75 pairs of full-siblings. All experimental pigs were healthy and not received antibiotic, probiotics or prebiotics during the period of experiment. Each pen housed 8–12 pigs. Male and female pigs were separately housed in different pens. Piglets were weaned at the age of 28 days, and then were raised in the nursery pen. As their body approached 30 kg, these pigs were transferred to the fattening house. The pigs that had a body weight greater than 100 kg would be slaughtered. Only in the fattening house, we installed the instrument of automatic feeding trough (Osborne Industries, USA) to measure phenotypic performances. Feed and water were available ad libitum in the stage of fattening between 30 kg (at the age of 70 ~ 90 days) and 100 kg body weight (at the age of 170 ~190 days). To avoid the effect of environmental adaptation of experimental pigs with automatic feeding trough, we used the phenotypic values obtained from day 100 to 160 (intermediate stage of measurement) for further analysis. |
| **Experimental procedures** | 7 | We collected the fecal samples from experimental pigs’ anus at the age of 140 days. After dipped in liquid nitrogen, all fecal samples were transferred into −80◦C freezer until use. Microbial DNA was extracted from feces with QIAamp Fast DNA Stool Mini Kit. The conserved primers 515F and 806R were used to amplify the V4 hypervariable region of 16S rRNA gene. PCRs were performed in 30 cycles at 60^◦^C of annealing temperature, and the products were separated by gel electrophoresis. After purification, the PCR products were used to construct the libraries, and then sequenced on a MiSeq platform (Illumina, USA) at the Beijing Genomics Institute (BGI, China). |
| **Experimental animals** | 8 | **a.** 280 commercial Duroc pigs (111 females and 169 males) **b.** The experimental pig cohort contained 24 pairs of half-siblings and 75 pairs of full-siblings **c.** fecal samples from experimental pigs’ anus at the age of 140 days. |
| **Housing and husbandry** | 9 | Each pen housed 8–12 pigs. Male and female pigs were separately housed in different pens. All experimental pigs were weaned at the age of 28 days and raised under the same feeding and management manners with the same commercial formula diet in man-control condition. Feed and water were available ad libitum in the stage of fattening. The commercial formula diet mainly contained corn, soybean meal, soybean oil, and calcium hydrophosphate, and consisted of 2,440 kJ digestible energy, 17% crude protein, 0.94% lysine, 0.9% calcium, 0.34% phosphorus, and 0.16% salt. |
| **Sample size** | 10 | A total of 280 fecal DNA samples were performed the 16S rRNA gene sequencing |
| **Allocating animals to experimental groups** | 11 | In this study, all experimental pigs were raised under the same feeding and management manners with same commercial formula diet. |
| **Experimental outcomes** | 12 | All experimental pigs were healthy during the phenotypic period. |
| **Statistical methods** | 13 | **a.** t-test was used to analyze the porcine phenotypic differences between different pens, sex and host genetics; **b.** Because of the non-normal distribution of the relative abundances of most bacterial taxa in the experimental pigs, a two-part model was applied to analyze the association of bacterial taxa with the traits of feeding behavior **c.** Correlation between the ADFI values and the relative abundances of KEGG pathways at the third class were implemented with MaAsLin.  d. PICRUST (v1.0.0) was applied to calculate the relative abundances of KEGG pathways according to the 16S rRNA gene sequences |
| **RESULTS** |  |  |
| **Baseline data** | 14 | To avoid the effect of environmental adaptation of experimental pigs with automatic feeding trough, we used the phenotypic values obtained from day 100 to 160 (intermediate stage of measurement) for further analysis. We collected the fecal samples from experimental pigs’ anus at the age of 140 days (body weight about 80~90 kg). |
| **Numbers analyzed** | 15 | A total of 280 pigs were phenotyped and had the 16S rRNA sequencing data |
| **Outcomes and estimation** | 16 | **a.** Pen had significant effect on phenotypes. The pigs in the same pen exhibited more similarity of appetite (*P* < 0.05) **b.** The *Prevotella*-predominant enterotype had a significantly higher ADFI value than the *Treponema*-enterotype (P = 0.01) **c.** A total of 34 OTUs were significantly associated with the ADFI in the two-part model analysis (FDR < 0.05). **d.** At the significance threshold of FDR < 0.05, a total of 16 predicted KEGG function terms showed significant associations with the ADFI by MaAsLin analysis. The ADFI-associated bacterial taxa contributed to the changes of predicted function capacities of gut microbiome. |
| **Adverse events** | 17 | None |
| **DISCUSSION** |  |  |
| **Interpretation/scientific implications** | 18 | [The present research showed that some bacteria producing SCFAs and lactic acid (e.g. Ruminococcaceae and Lactobacillus) might play an important role in suppressing porcine feed intake, while Prevotella could promote porcine feed intake and might be the keystone bacteria for host appetite control.](https://www.ncbi.nlm.nih.gov/pmc/articles/PMC2893951/table/pbio-1000412-t002/?report=objectonly#nt101) |
| **Generalisability/translation** | 19 | The results from this study give us an important cue that we can regulate pig appetite by modulating the gut microbiota. Furthermore, as pigs have been used as a biomedical model for human diseases, the identification of bacterial taxa involved in feeding behavior would also provide useful references for the prevention and treatment of human eating disorders. |
| **Funding** | 20 | This study was funded by National Natural Science Foundation of China (grant number 31472071 and 31702103). Hui Yang was supported by National Postdoctoral Program for Innovative Talents (No. BX201700102). The funding bodies had no role in the design of the study and collection, analysis, and interpretation of data and in writing this manuscript. |
|  |  |  |
